# Distinct Genes with Similar Functions Underlie Convergent Evolution in *Myotis* Bat Ecomorphs

**DOI:** 10.1093/molbev/msae165

**Published:** 2024-08-08

**Authors:** Ariadna E Morales, Frank T Burbrink, Marion Segall, Maria Meza, Chetan Munegowda, Paul W Webala, Bruce D Patterson, Vu Dinh Thong, Manuel Ruedi, Michael Hiller, Nancy B Simmons

**Affiliations:** Department of Mammalogy, Division of Vertebrate Zoology, American Museum of Natural History, New York, USA; Department of Herpetology, Division of Vertebrate Zoology, American Museum of Natural History, New York, USA; Centre for Translational Biodiversity Genomics, Frankfurt am Main, Hessen, Germany; Senckenberg Research Institute, Frankfurt am Main, Hessen, Germany; Faculty of Biosciences, Goethe-University, Frankfurt am Main, Hessen, Germany; Department of Herpetology, Division of Vertebrate Zoology, American Museum of Natural History, New York, USA; Department of Herpetology, Division of Vertebrate Zoology, American Museum of Natural History, New York, USA; Institut de Systématique, Evolution, Biodiversité (ISYEB), UMR 7205, Muséum National d’Histoire Naturelle, CNRS, SU, EPHE, UA, CP 50, Paris, France; Department of Life Sciences, The Natural History Museum, London SW7 5BD, UK; Department of Mammalogy, Division of Vertebrate Zoology, American Museum of Natural History, New York, USA; Escuela de Biología, Universidad Industrial de Santander, Bucaramanga, Santander, Colombia; Centre for Translational Biodiversity Genomics, Frankfurt am Main, Hessen, Germany; Senckenberg Research Institute, Frankfurt am Main, Hessen, Germany; Faculty of Biosciences, Goethe-University, Frankfurt am Main, Hessen, Germany; Department of Forestry and Wildlife Management, Maasai Mara University, Narok 20500, Kenya; Negaunee Integrative Research Center, Field Museum of Natural History, Chicago, USA; Institute of Ecology and Biological Resources, Vietnam Academy of Science and Technology, 18 Hoang Quoc Viet Road, Cau Giay District, Hanoi, Vietnam; Graduate University of Science and Technology, Vietnam Academy of Science and Technology, 18 Hoang Quoc Viet Road, Cau Giay District, Hanoi, Vietnam; Department of Mammalogy and Ornithology, Natural History Museum of Geneva, Geneva 1208, Switzerland; Centre for Translational Biodiversity Genomics, Frankfurt am Main, Hessen, Germany; Senckenberg Research Institute, Frankfurt am Main, Hessen, Germany; Faculty of Biosciences, Goethe-University, Frankfurt am Main, Hessen, Germany; Department of Mammalogy, Division of Vertebrate Zoology, American Museum of Natural History, New York, USA

**Keywords:** bats, convergent evolution, comparative genomics, ecomorphs

## Abstract

Convergence offers an opportunity to explore to what extent evolution can be predictable when genomic composition and environmental triggers are similar. Here, we present an emergent model system to study convergent evolution in nature in a mammalian group, the bat genus *Myotis*. Three foraging strategies—gleaning, trawling, and aerial hawking, each characterized by different sets of phenotypic features—have evolved independently multiple times in different biogeographic regions in isolation for millions of years. To investigate the genomic basis of convergence and explore the functional genomic changes linked to ecomorphological convergence, we sequenced and annotated 17 new genomes and screened 16,426 genes for positive selection and associations between relative evolutionary rates and foraging strategies across 30 bat species representing all *Myotis* ecomorphs across geographic regions as well as among sister groups. We identify genomic changes that describe both phylogenetic and ecomorphological trends. We infer that colonization of new environments may have first required changes in genes linked to hearing sensory perception, followed by changes linked to fecundity and development, metabolism of carbohydrates, and heme degradation. These changes may be linked to prey acquisition and digestion and match phylogenetic trends. Our findings also suggest that the repeated evolution of ecomorphs does not always involve changes in the same genes but rather in genes with the same molecular functions such as developmental and cellular processes.

## Introduction

Convergent evolution occurs when two or more lineages independently evolve the same adaptations, often under similar selective pressures or in similar environments. Trait convergence (phenotypic and/or ecological) is common in nature and found among all groups of organisms (Conte et al. 2012; Rajakumar et al. 2012; Sackton and Clark 2019). Natural selection has been proposed as the driver of convergence because it is unlikely that the same trait would evolve randomly more than once (Schluter and Nagel 1995). Thus, the repetitive evolution of a trait may imply selective changes in the same coding or noncoding genic regions even across different taxa. Other genomic changes, such as gene losses due to inactivating mutations (Sharma et al. 2018), gene duplications (Amoutzias et al. 2004), parallel amino acid changes (Lee et al. 2018), and gene transfer (Xu et al. 2020), can have important implications for convergent adaptations. For example, Hox genes control embryonic development, and rearrangements in such genes can result in body plans with similar structures and functions, such as legs in insects and vertebrates (Pearson et al. 2005) or pseudo-thumbs in carnivores (Fang et al. 2023). Conte et al. (2012) estimated the mean probability of reusing the same genes in reported cases of convergent evolution of phenotypes in natural populations of the same species and estimated that gene reuse is more common among recently diverged lineages than those that are more distantly related. Even though the studies explored by Conte et al. (2012) targeted specific loci known to be linked to particular traits, their implications suggest a way forward to design and test hypotheses regarding the predictability of trait evolution at genomic scales and to explore in detail to what extent repeated evolution implies structural changes in the same genes or in genes with the same functions.

In the last decade, genomic data and methods have provided novel perspectives on studies of convergent evolution. For example, the genomic basis of convergent marine adaptations in mammals (Foote et al. 2015), limb loss in reptiles (Roscito et al. 2022; Ovchinnikov et al. 2023), and caecilians (Roscito et al. 2022; Ovchinnikov et al. 2023) and convergent flight loss in birds (Sackton et al. 2019) was investigated by performing comparative analyses of coding genes and regulatory elements. In marine mammals, convergent amino acid substitutions in genes under positive selection are relatively common; however, adaptive molecular convergence linked to convergent phenotypes to aquatic life is comparatively less frequent (Foote et al. 2015). In reptiles, independent changes in individual limb regulatory elements, rather than shared sequence divergence or transcription-binding factor sites, likely have led multiple evolutionary paths to limb loss (Roscito et al. 2022; Ovchinnikov et al. 2023). In birds, convergent evolution of regulatory regions, more so than protein-coding genes, is commonly found among developmental pathways associated with independent losses of flight (Sackton et al. 2019). At shallower evolutionary time scales, studies combining whole-genome datasets with host-transplant experiments of populations of stick insects undergoing parallel speciation, a type of convergent evolution, identified unique genomic regions of high divergence between populations. Additionally, parallel changes occurred in coding genes suggesting repeated and independent evolutionary changes across ecotypes (Soria-Carrasco et al. 2014). As noted by several authors, convergent evolution implies that different species independently evolve similar traits from different ancestral starting points, potentially driven by similar selective pressures and controlled by nonhomologous genes (e.g. Desutter-Grandcolas et al. 2007; Lecointre and Le Guyader 2007). In contrast, parallel evolution involves similar traits evolving independently in species that share a more recent common ancestor, often following similar genetic changes in homologous genes. Prematurely labeling the evolution of similar traits as parallel could introduce the assumption of changes in homologous genes, potentially leading to confusion. We thus prefer to use the term convergence.

**Fig. 1.**
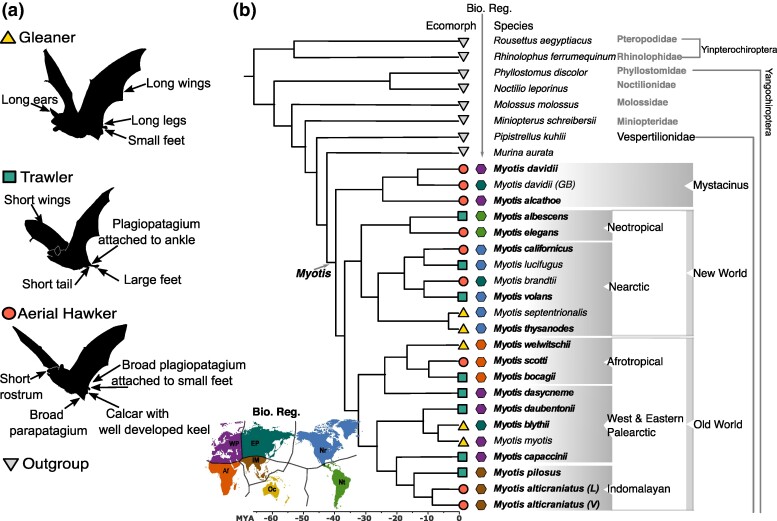
Morphological features and phylogenetic relationships among *Myotis* bats. a) Silhouette and phenotypic description of the three *Myotis* ecomorphs as noted in Findley (1972), Koopman (1994), and Morales et al. (2019). b) Phylogenetic tree used for genomic screening with 30 bat species. At the terminal branches, symbols in the first column indicate foraging strategy as in a), hexagons in the second row indicate biogeographic regions and species distribution colored following the map inset at the lower left, and labeled as Nearctic (Nr), Western Palearctic (WP), Eastern Palearctic (EP), Afrotropical (Af), Neotropical (Nt), Indomalayan (IM), and Oceanian (Oc). Bold text indicates new genomes generated in this study. GB = Genome of *M. davidii* obtained from GenBank. Sample from *M. alticraniatus* collected in Vietnam (V), or Laos (L). Branch lengths represent millions of years before the present (mya) with a scale at the bottom. Bat silhouettes modified from Table 1 of Morales et al. (2019).

Nonetheless, studying convergent evolution in nature has practical challenges, particularly in vertebrates, which often have long generation times and where most species are difficult or expensive to maintain in captivity (Li et al. 2016). Despite significant efforts, a better understanding of the genomic and molecular basis of repeated convergent evolutionary changes under natural conditions in long-lived species is needed. The biology of many taxa limits the feasibility of testing hypotheses that might require experiments involving genetic crosses and translocation of populations. Therefore, many predictions must be addressed with alternative experimental designs, such as using museum specimens coupled with genome-wide approaches (e.g. Moreira and Smith 2023). The bat genus *Myotis* provides a valuable new model system for disentangling the genomic mechanisms underlying convergent ecological and phenotypic adaptations that evolved under natural conditions over comparatively long time scales. *Myotis* are the most species-rich genus of bats (139 species; Simmons and Cirranello 2024) within the family Vespertilionidae and among the order Chiroptera. *Myotis* is among the few non-commensal mammalian genera that have colonized all biogeographic regions and adapted to numerous biomes. However, phenotypic variation among species is restricted to three foraging strategies that are each thought to have repeatedly evolved several times (Ruedi and Mayer 2001; Morales et al. 2019). These foraging strategies were originally associated with subgenera based on morphology and feeding modalities: foliage gleaners (subgenus *Myotis*), trawlers (subgenus *Leucone*), and aerial hawkers (subgenus *Selysius*; Findley 1972; Morales et al. 2019). The main morphological features associated with subgenera were described based on a data set of 48 cranial and postcranial characters focused on the size and shape of the head, ears, hind limbs, wings, and the number of teeth (Findley 1972; Koopman 1994; Table 1; Fig. 1a). Interestingly, subsequent molecular phylogenetic analyses showed that these feeding strategies and morphologies each evolved several times and that the three recognized subgenera were not reciprocally monophyletic but instead represented ecomorphs (Ruedi and Mayer 2001; Ruedi et al. 2013; Morales et al. 2019). While the transition between trawling and aerial hawking ecomorphs appears to have been more common in recent evolutionary scales (e.g. hundreds of thousands of years to a few million years), foliage gleaners have evolved at least three times in Eurasia (Palearctic and Indomalayan clades), Africa (Afrotropical clade), and North America (Nearctic clade), in each case having been isolated for several millions of years (Fig. 1b; Ruedi and Mayer 2001; Ruedi et al. 2013; Morales et al. 2019). The repetitive evolution of convergent phenotypes in *Myotis* in different environments over several millions of years offers an exceptional opportunity to study the genomic and ecological mechanisms of convergence without controlled lab experiments or reintroductions.

**Table 1:** Morphological descriptions of ecomorphs in *Myotis* bats (Findley 1972; Koopman 1994; Morales et al. 2019)

Foraging modality	Main phenotypic features
Gleaners from foliage and other surfaces (soil, grass, and leaves, among others)	Relative to body size, large head with narrow braincase, long legs, long wings, and small feet. Relative to size of head, long ears, and long jaws.
Trawlers or water surface foragers	Relative to body size, short hind limbs, but elongated feet. Parapatagium attached to the ankle. Relative to size of head, wide skull and extended toothrow.
Aerial hawkers or netters	Relative to body size, small limbs with broad parapatagium with lower attachment of the wing membrane to the leg. Relative to size of head, short rostrum, and short jaws. Rounded skull. Broad parapatagium. Calcar with a well-developed keel.

See silhouette in Fig. 1a

**Fig. 2.**
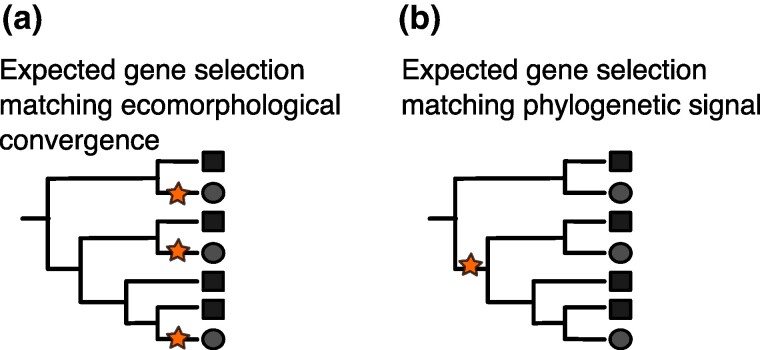
Expected patterns of gene selection on branches indicating ecomorphological convergence a) or phylogenetic signal b). Stars represent genes selected on specific branches.

Here, we offer a model for genomic investigations into ecological and morphological convergence in nature, focusing on the bat *Myotis* genus. Our aims are (i) to explore the genetic changes associated with positive selection, evolutionary rate shifts, and protein convergence that are associated with convergent foraging strategies (Fig. 2a) or phylogenetic signal (Fig. 2b) and (ii) to understand whether observable trait similarities correlate with homologous or nonhomologous genes. Using a comparative genomic approach, we sequenced and annotated genomes from representative *Myotis* species across ecomorphs and biogeographic regions. We explored whether orthologous genes are consistently under positive selection in species that show similar foraging strategies or live in similar environments (biogeographic regions). Additionally, we tested the hypothesis of molecular convergence linked to repeated evolution of ecomorphs by examining convergence in evolutionary rates among foraging groups and assessing protein evolution rates within species pairs of the same foraging group by looking at changes from potentially any ancestral state (ancestral ecomorphs) to specific derived states that we observe in the present (gleaners, aerial hawkers, and trawlers). We explored the probability of gene reuse linked to phenotypic evolution by examining the correlation between species divergence time and the proportion of shared positively selected genes or genes with significant protein convergence rates within each ecomorph. Taken together, our results indicate that repetitive evolution of traits does not involve changes in the same genic regions, but rather in genes with similar functions.

## Results

### Myotis Genome Diversity

To identify genomic changes linked to phenotypic convergence in *Myotis*, first we sequenced and annotated 17 genomes of species representing the 3 *Myotis* foraging strategies across all biogeographic regions (supplementary table S1, Supplementary Material online). We applied a reference-guided assembly approach, using as a reference the most complete and contiguous *Myotis* genome generated thus far (*Myotis myotis*, Jebb et al. 2020). The scaffolds of our assemblies are comparable to those of *M. myotis* where the average N50 is 94.45 Mb (supplementary table S2, Supplementary Material online). We used TOGA, a tool to infer orthologs from genome alignments, to generate gene annotations for all our genomes representing 46,734 transcripts from 19,306 genes. TOGA is a reference-based annotation method; thus, we used as the input reference the highly complete annotation of the human genome (hg38). Even though the divergence time between human and bats is estimated to be around 70 to 95 mya (Upham et al. 2019; Álvarez-Carretero et al. 2022; Foley et al. 2023), the human genome annotation is the most comprehensive to date for a mammalian species and therefore provides an excellent source of reference transcripts. In our annotations, the BUSCO scores for the number of complete genes range from 6,808 (73.9% of mammalia_odb10 for *Myotis dasycneme*) to 8,975 (97.28% of mammalia_odb10 for *Myotis pilosus*; supplementary fig. S1, Supplementary Material online). In addition, TOGA provides an alternative metric for genome annotation quality based on the number of genes with intact reading frames, referring to those genes that lack inactivating mutations or missing sequences in the 80% central portion of the gene. Such a metric in our genomes ranges from 6,836 (*M. dasycneme*) to 16,528 (*M. pilosus*) within a set of 18,430 conserved genes across mammals (supplementary fig. S2, Supplementary Material online).

### Genetic Changes Linked to Colonization of New Biogeographic Regions

To explore if there are patterns of gene selection in *Myotis* that can be linked to adaptations of colonizing biogeographic regions (indicating phylogenetic signal) or foraging groups (indicating ecomorphological convergence), we performed a comprehensive genomic screen for all the branches in our phylogenetic tree using an adaptive branch-site random effects likelihood method (aBSREL; Smith et al. 2015). We included 16,426 single-copy ortholog genes from 30 bat genomes, including 22 *Myotis* (Table 2; 17 newly sequenced and annotated here) representing all ecomorphs and biogeographic regions, and a total of 8 outgroup bat species: 2 species representing sister genera within the family Vespertilionidae (the family to which *Myotis* belongs), 4 species representing sister families within the suborder Yangochiroptera (Miniopteridae, Molossidae, Noctilionidae, and Phyllostomidae), and 2 species representing different families from the sister suborder Yinpterochiroptera (Pteropodidae and Rhinolophidae) (Fig. 1b; supplementary table S1, Supplementary Material online).

**Table 2:** Number of *Myotis* specimens and genomes used for gene selection screens

Genomes	Nr	Nt	Af	WP	EP	IM	O	Total
Trawler	2	1	1	2	1	1	0	8
Gleaner	2	-	1	1	1	0	-	5
Aerial netter	1	1	1	2	2	2	-	9
Total	5	2	3	5	4	3	0	22

Columns represent biogeographic regions as Nearctic (Nr), Neotropical (Nt), Afrotropical (Af), Western Palearctic (WP), Eastern Palearctic (EP), Indomalayan (IM), and Oceanian (Oc), and rows represent foraging strategies. If cell value is “-”, it means such foraging strategy has not been reported in the biogeographic region.

**Fig. 3.**
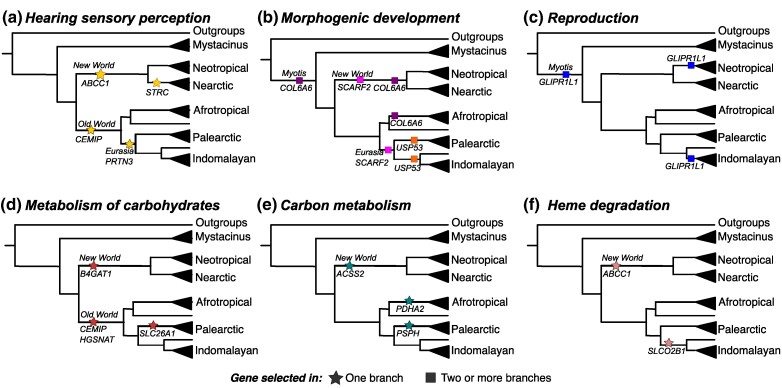
Relevant genes under positive selection in *Myotis* branches that correspond to biogeographic regions. Clades were collapsed (shown in a triangle) per biogeographic regions described in Fig. 1b, except for those species with geographic distributions nested in different clades (such as the Palearctic *M. dasycneme* in the clade of Afrotropical species and *M. capaccinii* in the clade of Indomalayan species; however, *M. brandtii* was nested in the Nearctic clade). Squares indicate genes selected more than once in different branches. Stars represent genes under positive selection in only one branch. Comprehensive list of genes under selection in supplementary table S3, Supplementary Material online.

We identified 96 genes under positive selection only in *Myotis* branches, which correspond to the phylogenetic structure matching the colonization of major biogeographic areas (Fig. 3; supplementary table S3, Supplementary Material online). For example, stem branches for such clades or biogeographic groups represent the divergence between New World and Old World clades that indicate the colonization of *Myotis* lineages from Eurasia to the Americas (Fig. 1b and Fig. 3). Genes under selection in such branches are involved in hearing sensory perception, morphogenic development, reproduction, and metabolic functions (Fig. 3).

First, regarding sensory perception, we identified genes under positive selection linked to deafness or hearing impairment (Fig. 3a). For example, CEMIP (or KIAA1199) is under selection on the stem branch of the Old World *Myotis* clade, which includes species from Europe, Asia, and Africa. CEMIP is expressed in the cochlea and has a key role in maintenance of epithelial cells; mutations in this gene are linked to loss hearing in humans (Abe et al. 2003; Yoshida et al. 2013; Hosoya et al. 2016). PRTN3 is under selection on the stem branch of the Palearctic and Indomalayan clades (including mainly species from Eurasia). PRTN3 is usually expressed in the middle ear canal, and a lack of expression can cause chronic disease leading to hearing loss (Narayan et al. 2021). In parallel, ABCC1 is under selection on the stem branch of the New World clade, which includes species from the Americas. This gene is expressed in the cochlea auditory nerve that has an important role in extruding pumps for maintaining the cochlea function, and substitutions in particular residues are linked to hereditary deafness (Li et al. 2019). STRC is under selection on the stem branch of the Nearctic clade, which includes species from North America. This gene is linked to the hair bundle of the sensory hair cells in the inner ear, and autosomal mutations are linked to deafness and infertility (Zhang et al. 2007; Nishio and Usami 2022).

Second, regarding genes under positive selection associated with morphogenic development (Fig. 3b), which holds implications for understanding adaptation to diverse environments, SCARF2 is under selection in the Palearctic and Indomalayan clades (corresponding to the Eurasia group) and New World *Myotis* clades. Mutations in this gene in humans are involved in underdeveloped eyelids and jaw bones and are also linked to phenotypes such as long and bent fingers, cleft palate, and other bone abnormalities (Hwang et al. 2005; Anastasio et al. 2010). COL6A6 is under selection in the stem *Myotis* branch and in the stem branches of the Afrotropical and New World *Myotis* clades, which include species from southern Europe and Africa and the Americas. COL6A6 is a gene that in humans has a key role in the developmental transition from embryos to fetuses, such as ossification, skeletal muscle development, extracellular matrix organization, cardiovascular system, erythrocyte differentiation, and neuronal system (Teixeira et al. 2021). In addition, USP53 was found to be under selection in the Indomalayan and Palearctic clades. USP53 is a gene that encodes a tight junction-associated protein essential for the survival of auditory hair cells and normal hearing in mice, possibly by modulating the barrier properties and mechanical stability of tight junctions (Kazmierczak et al. 2015).

Third, regarding genes under positive selection associated with the reproductive system (Fig. 3c), GLIPR1L1 (Gaikwad et al. 2019), a gene that plays an important role in the binding between sperm and oocytes, was found to be under selection in the stem *Myotis* branch and in the branches of the Indomalayan and Neotropical clades, which include species from Southeast Asia and South America, respectively.

Finally, regarding metabolic adaptations, we identified genes under positive selection involved in metabolism of carbohydrates, carbon metabolism, and heme degradation in stem branches for Old World, New World, Nearctic, Neotropical, Afrotropical, Palearctic, and Indomalayan clades (Fig. 3d to f). For example, genes involved in the metabolism of carbohydrates (e.g. glycosaminoglycan) that have structural roles in connective tissue, cartilage, bone, and blood vessels were found to be under selection in the branches leading to Old World *Myotis* (CEMIP and HGSNAT), New World *Myotis* (B4GAT1), and the Palearctic clade (SLC26A1; Fig. 3d). Genes involved in carbon metabolism—an essential process in the integration of the nutritional status of cells and essential in cellular physiology—were found to be under selection in New World *Myotis* (ACSS2), Afrotropical (PDHA2), and Palearctic clades (PSPH; Fig. 3e). In addition, genes linked to metabolic pathways of heme degradation—a key process in the recycling of iron, antioxidant defense, and cellular signaling (Otterbein et al. 2003; Wilks and Heinzl 2014)—were found to be under selection in New World *Myotis* (ABCC1) and the Indomalayan clade (SLCO2B1; Fig. 3f).

### Genetic Changes Linked to Ecomorphological Convergence

**Fig. 4.**
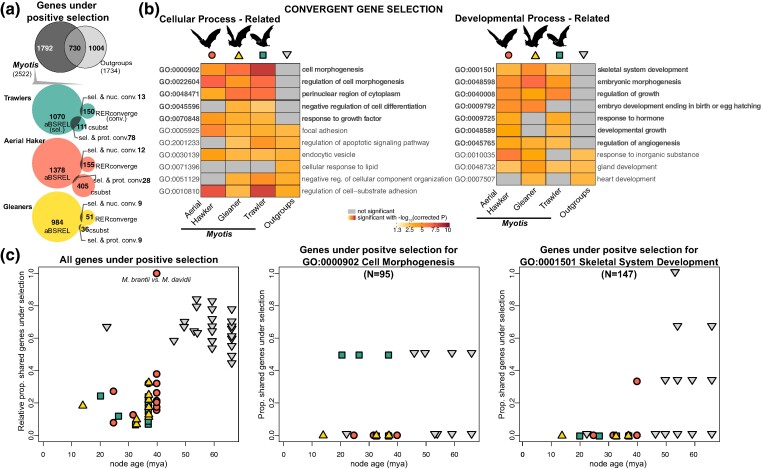
Patterns of convergent gene selection linked to foraging strategies in *Myotis* and outgroup bats. a) Venn diagram showing the count of genes under positive selection, as identified by the aBSREL analysis. At the bottom, colored circles illustrate the proportion of genes under positive selection that overlap with genes with convergent evolutionary rates linked to each foraging strategy, as determined by the RERconverge and csubst analyses. b) Functional enrichments for genes under positive selection across each foraging strategy and non-*Myotis* outgroup species (columns). GOs associated with cellular and developmental processes are depicted (rows). GOs with statistically significant enrichments are highlighted in color, while nonsignificant values are shown in gray. GO terms with significant values across *Myotis* ecomorphs, but not in outgroups, are bolded. c) Regression analysis showing the relationship between the relative proportion of shared genes under positive selection (gene reuse) and node age. The analysis is based on all genes under selection for each species pair and relevant GOs, including genes under selection in *Myotis* pairs versus non-*Myotis* pairs. Relative values were calculated by the maximum number of observed shared genes between *M. davidii* and *M. brandtii* (103 genes under selection). Symbols indicate foraging strategy as trawler (square), aerial hawker (circle), or gleaner (triangle).

To identify genes under positive selection associated with foraging groups, indicating ecomorphological convergence, we compiled information on shared patterns of gene selection from species exhibiting the same foraging strategies using the results of the aBSREL analyses. We focused on extant species (terminal branches in our tree) where observable phenotypes can be studied in living species. Out of 16,426 genes screened, we identified 984, 1,378, and 1,070 genes under selection in at least one species within each of the phenotypic groups identified as gleaners, aerial hawkers, and trawlers respectively (Fig. 4a; supplementary table S4, Supplementary Material online). Among these genes, around 70% to 80% were under positive selection only in species that show the same foraging phenotype, while the remaining 20% to 30% were under selection in more than one foraging group (supplementary table S3, Supplementary Material online). However, we found that lineage-specific genes under selection within species of the same foraging strategies shared similar Gene Ontology (GO) annotations, particularly those associated with cellular and developmental processes such as cell morphogenesis, regulation of cell differentiation, and response to growth factors (Fig. 4b). This similarity in functional associations was consistent within *Myotis* ecomorphs but differed from those observed in non-*Myotis* outgroups, as revealed by gene enrichment analyses (Fig. 4b). To ensure the robustness of our findings regarding cellular and developmental processes, we conducted a randomized gene enrichment test. This test involved selecting genes under positive selection at random for *Myotis* ecomorphs and non-*Myotis* species and assessing their functional annotation. Our results indicate that the distinctive pattern observed in *Myotis* in terms of cell morphogenesis, regulation of cell differentiation, and response to growth factors is not a product of background evolution common to all bats. Rather, it reflects specific functional associations within the ecomorphs (supplementary fig. S3, Supplementary Material online). Moreover, we confirmed that the patterns observed are not driven by a single species, but rather consistent across species when they are analyzed independently (supplementary fig. S4, Supplementary Material online).

To investigate the potential for “gene reuse” in of the evolution convergent phenotypes associated with divergence time—where changes in the same genes are associated with convergent traits among closely related lineages, as noted by Conte et al. (2012)—we examined whether there is a significant correlation between the proportion of shared genes under selection and the divergence time (node age) for each species pair with the same *Myotis* ecomorph. We first tested a model that included all shared genes under positive selection for given pairs. Contrary to the expected results reported by Conte et al. (2012) for intraspecific gene reuse, we found a significant, although low, positive correlation between the proportion of all shared genes under selection and the divergence time (*R*^2^ = 0.0702, *P* < 0.0001), indicating that the number of shared genes increases with node age (Fig. 4c). Then, to estimate gene reuse linked to phenotypic evolution, we selected sets of gene candidates associated with cell morphogenesis (GO:000090) and skeletal system development (GO:0001501) given that these terms were most significant in the gene enrichment analyses for cellular and developmental processes within *Myotis* species but not in the outgroups. For genes annotated as part of cell morphogenesis, there was a nonsignificant correlation between the proportion of shared genes under selection and the divergence time (*R*^2^ = 0.0017, *P* = 0.50288). Although the correlation was not significant, the negative trend was expected according to Conte et al. (2012). However, for genes annotated as part of skeletal system development, we found a significant and positive correlation, though with weak effect size, between the proportion of shared genes under selection and the divergence time (*R*^2^ = 0.0719, *P* < 0.0001). This may indicate that the number of shared genes increases with node age. We noted that the proportion of shared genes between *Myotis* lineages was lower than expected compared to the outgroup species, supporting the idea that convergent evolution among ecomorphs in *Myotis* involves different genes with similar functions, but gene reuse has been relatively rare.

### Assessing Convergent Evolutionary Rates

**Fig. 5.**
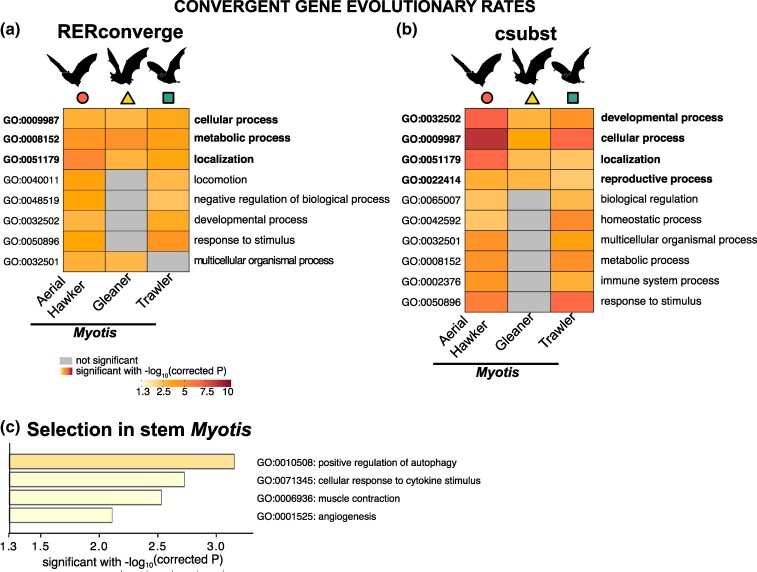
Functional enrichments for genes with convergent evolutionary rates in *Myotis* bats. a) Genes exhibiting a significant correlation of relative evolutionary rates linked to foraging strategies in *Myotis* ecomorphs (columns), as identified by the RERconverge analysis. b) Genes exhibiting convergent rates of protein evolution linked to foraging strategies in *Myotis* ecomorphs (columns), as identified by the csubst analysis. GO associated with biological processes are depicted in rows for A and B. Significant enrichments are highlighted in color, while non-significant values are displayed in gray. The analyses of RERconverge and csubst focus on species with observed convergent ecomorphs, thus outgroup species lacking these phenotypes cannot be included. c) Gene set enrichment results for 39 genes under positive selection only in stem *Myotis* branch.

Positive selection in genes with the same functions can lead to equivalent evolutionary shifts in nucleotide substitution and protein convergence; thus, we next tested (i) if there is a correlation between the relative evolutionary rates of single-copy ortholog genes and convergence within each foraging strategy and (ii) convergence in rate between branch pairs at the protein level. First, we used a method that combines data transformation and weighted regressions while correcting for heteroscedasticity in gene evolutionary rates and accounting for phylogenetic relatedness, implemented in RERconverge. We identified a smaller set of genes with significant signal for convergence in relative evolutionary rates linked to a foraging phenotype (supplementary table S6, Supplementary Material online). For example, 51, 155, and 150 genes within gleaners, aerial hawkers, and trawlers, respectively, showed significant correlations between their relative nucleotide substitution rate and the branches that correspond to a particular foraging strategy (Fig. 4a). Similar to the enrichment results for genes under selection, genes with convergent relative evolutionary rates showed the same GO annotations such as cellular and metabolic processes and localization of substances or other cellular entities. This pattern was consistent among the three foraging groups (Fig. 5a; supplementary table S7, Supplementary Material online). Second, using csubst (Fukushima and Pollock 2023), a method that incorporates a metric (ωc) which measures error-corrected convergence rates between branch pairs at the protein level, we identified 36, 405, and 111 genes within gleaners, aerial hawkers, and trawlers, respectively, that showed significant ωc > 3 values indicating convergence for any ancestral state transitioning to specific derived states observed in our terminal branches with known foraging strategies (supplementary table S8, Supplementary Material online). Enrichment tests with these genes confirmed that species within each *Myotis* ecomorphs showed significant convergence across genes linked to cellular and localization processes (supplementary table S9, Supplementary Material online). In addition, we detected protein convergence in genes linked to developmental and reproductive processes (Fig. 5b).

To identify genes with both signatures of selection and convergent rates of nucleotide substitution and protein evolution, we cross-referenced our results from aBSREL, RERconverge, and csubst. We found genes among species in each foraging phenotype that simultaneously exhibited both significant signals of selection and convergent relative evolutionary rates as detected by RERconverge or csubst (Fig. 5a). We did not find overlapping genes between results from RERconverge or csubst, likely due to methodological differences. For instance, RERconverge uses branch lengths, whereas csubst examines amino acid substitutions in branch pairs, employing an ancestral sequence reconstruction approach. Nevertheless, the number of overlapping genes with the aBSREL results represents a small sample size yielding significant *P*-values in gene set enrichment analyses. However, in gleaners, such genes under selection and with convergent rates are mainly part of pathways involved in signal transduction and protein localization; in aerial hawkers, such genes are part of the adaptive immune system and programmed cell death pathways; and in trawlers, such genes are part of the innate immune system and hemostasis.

### Genes Under Positive Selection in Stem Myotis

Finally, to explore which genes may have contributed to the successful radiation of the *Myotis* clade, we focused on the genes under selection only in the stem branch. We identified 39 genes under selection only in the stem *Myotis* (supplementary table S3, Supplementary Material online). These genes are significantly enriched in GO for Biological Processes (GO: 0008150) such as regulation of autophagy (e.g. DHX33, VDAC1, STING1, and MTDH) which promote degradation and reuse of damaged cellular components; cellular response to cytokine stimulus (e.g. FGF23, OASL, LIFR, STING1, and PLP2) that involve key processes to regulate inflammatory responses; muscle contraction (e.g. CCDC78, CALD1, and MYLK2), essential for locomotor activity; and angiogenesis (e.g. DLL4, CALD1, and ARHGAP22), a process that involves the generation of new blood vessels and is essential for wound healing (Fig. 5c).

## Discussion

The evolutionary history of *Myotis* bats provides a useful example for studying the genomic features driving convergent phenotypic adaptations. Following previous findings that *Myotis* bats show a strong signal of phenotypic convergence across multiple clades (Ruedi and Mayer 2001; Ruedi et al. 2013; Morales et al. 2019), we performed genomic screens to explore whether gene selection changes are associated with similar phenotypes and ecological strategies, or instead reflect phylogenetic relatedness. We identified genetic changes that support both ecomorphological and phylogenetic patterns. In the context of ecomorphological convergence, we might expect to find the same selection patterns in genes occurring in species with similar phenotypes (Conte et al. 2012). However, our results indicated this was not the case in *Myotis* bats. Our study revealed that genetic changes associated with positive selection or convergent substitution rates occur in regions annotated with similar functions, particularly at the level of GO but not in the same genes. Moreover, we observed unexpected correlations between shared gene selection and divergence time among bat ecomorphs. In contrast to previous expectations (Conte et al. 2012), we identified a low yet significant positive correlation between the proportion of shared genes under positive selection and divergence time, suggesting the frequency of convergent gene selection is not correlated with divergence time between ecomorphs. The number of positively selected genes associated with cell morphogenesis did not show significant correlations with divergence time, while those linked to skeletal system development exhibited a significant positive correlation with divergence time. Further research is needed to understand the specific genetic mechanisms underlying phenotypic convergence linked to gene reuse in this lineage and the timing of changes leading to different foraging strategies. We acknowledge the importance of ancestral ecomorph reconstructions for confidently associating positive selection with phenotype evolution. However, the challenge lies in accurately inferring ancestral ecomorphs with a partial dataset. While our dataset represents the largest collection of assembled and annotated genomes for the genus *Myotis*, it comprises ∼15% of the extant species (supplementary fig. S5, Supplementary Material online). Therefore, our interpretations of genetic changes linked to convergent phenotypes have focused on extant taxa and observable phenotypes in living species, strategically selected to represent all ecomorphs and biogeographic regions where *Myotis* species are found. We emphasize the need for future research to expand genomic sampling encompassing a more comprehensive representation of *Myotis* species, which will enhance the accuracy of ancestral reconstructions and improve our understanding of convergent evolution in this genus. Our findings highlight the complex dynamics of convergent evolution in extant *Myotis* bats, where gene reuse appears to be rare despite functional similarities. Notably, we found different genes with selection patterns and convergent relative evolutionary rates linked to the same biological functions (cellular, developmental, and metabolic processes). Our results indicate that convergent gene selection or substitution rates have acted at a higher organizational level, affecting gene pathways instead of individual genes. Therefore, commonalities of these feeding requirements have led to convergence of ecological and morphological functions.

When considering genetic changes associated with phylogenetic patterns, as expected, in our analysis we found genes under positive selection on branches that match biogeographic splits and correspond to phylogenetic groups. Such genes are mainly linked to hearing sensory perception, morphogenic development, reproduction, metabolism of carbohydrates and carbon, and heme degradation. Based on the known divergence time of branches under selection, we can assume that some changes happened earlier than others. For example, the selection signal for hearing genes on branches mirroring colonization of new biogeographic regions suggests that *Myotis* lineages first adapted to environmental pressures linked to spatial orientation. Subsequent adaptations seem to have been linked to reproductive barriers and behavioral and phenotypic adaptations for prey acquisition, including metabolic changes for digestion. It is hypothesized that in bats, laryngeal echolocation evolved first for spatial orientation and only later was modified in various ways to facilitate different prey acquisition strategies (Simmons and Geisler 1998; Schnitzler et al. 2003). Foraging ecology and audition are thus intricately interrelated and interdependent (Neuweiler 1989). All bats have to perform several tasks when searching for food, and at least three tasks—spatial orientation, habitat recognition, and food finding—often have to be performed in parallel (Denzinger and Schnitzler 2013). Therefore, species that forage in similar habitats are expected to share sensory system characteristics, particularly in the design of echolocation call signals to solve similar orientation tasks (Schnitzler et al. 2003). In *Myotis*, echolocation call structures are very similar among species and rarely provide sufficient information to differentiate species (Parsons and Jones 2000). This reinforces the idea that bats across different continents have evolved similar traits to acquire food. But after prey are acquired, they have to be digested, and thus, metabolic adaptations also come into play. Bats with similar foraging strategies may obtain similar prey types with similar nutrient, protein, and fat content; therefore, it is expected that similar physiological and metabolic adaptations are required for digestion. This may explain why genes linked to particular metabolic adaptations are under selection in species with similar foraging strategies.

In addition, we identified genes under positive selection only in the stem *Myotis* branch which are linked to muscle contraction, regulation of cell damage repair, generation of new blood vessels, wound healing, and inflammation. A common link among these processes is growth factors—a group of proteins, considered a subset of cytokines, that play crucial roles in regulating cellular processes such as growth, proliferation, differentiation, and survival, inflammation, and tissue repair (Meyer-Ingold 1993). Such processes are key to offsetting the highly energetic demands associated with powered flight (Shen et al. 2010). However, all bats can fly and have a monophyletic origin (Gunnell and Simmons 2005; Tsagkogeorga et al. 2013), so we expect that such flight-related adaptations evolved earlier. Nonetheless, within bats, Vespertilionidae is the most species-rich family, and *Myotis* is the most species-rich genus of bats, leading to questions about just what made them so successful and diverse. Additionally, the cryptic swarming behavior in *Myotis* bats may play a role in promoting introgression of immune-related genes by bringing individuals from different species into close proximity during mating (Foley et al. 2024), leading to genetic exchange as a potential source of species diversification. Moreover, the genome of vespertilionid bats includes higher transposable element (TE) content compared to other bat families and mammalian orders (Osmanski et al. 2023; Paulat et al. 2023). While TEs might regulate certain genes under selection in *Myotis* lineages and adaptive introgression of immune-related genes may contribute to speciation, we hypothesize that these genes could have provided an evolutionary advantage contributing to the diversity of the *Myotis* genus. However, this hypothesis requires thorough testing. The changes identified in genes under selection in *Myotis* offer a guide to explore the regulatory processes that may lead to more diverse groups since regulatory regions are particularly influential in modulating gene expression and phenotype variation, making them vital targets for future research on convergent evolution. Further gene regulatory, gene expression, and genome-wide association analyses at a population level can provide valuable insights into the genetic mechanism linked to *Myotis* species diversification and morphological convergence (Lamichhaney et al. 2019).

Here, our comparative genomic screen sheds light on the natural genomic mechanisms of convergent evolution by focusing on such a group of long-lived mammals, a diverse group of bat species that have been isolated for millions of years yet look and behave very similarly. We found a positive correlation between the proportion of shared genes under positive selection and divergence time, indicating an increase in gene reuse throughout evolutionary history, independent of repeated phenotypic evolution. Our results underscore the complex genetic dynamics of convergent evolution in *Myotis* bats, where the recurrence of genes under selection seems relatively rare despite shared functional characteristics. This study provides evidence that repetitive evolution of a trait does not always imply selection in the same genic regions across different taxa, but rather selection in genes with similar functions.

## Methods

### Sampling for Genomic Analyses

We analyzed 30 bat genomes, including 22 *Myotis* (Table 2), of which 17 were generated for this study and the rest were previously published (supplementary table S1, Supplementary Material online). Our sampling includes 20 *Myotis* species representing all ecomorphs described and all biogeographic regions (Table 1). The biogeographic history of *Myotis* is complex, in part, due to the successful colonization and recolonization of multiple environments (Ruedi et al. 2013; Morales et al. 2019). While most biogeographic groups represent monophyletic groups, there are notable examples of species not nested in such groups. For example, the Indomalayan and Afrotropical clades include species found outside the expected geographic distributions and in Western Palearctic regions (Fig. 1b). Therefore, our sampling includes some of these taxa as they represent key lineages to understand the complex evolutionary history of this group and identify genes potentially linked to ecomorphological convergence despite phylogenetic relatedness. In addition, we included outgroups from two sister genera within the same family (Vespertilionidae, *Murina aurata feae* and *Pipistrellus kuhlii*), four additional families from Yangochiroptera (Miniopteridae, *Miniopterus schreibersii*; Molossidae, *Molossus molossus*; Noctilionidae, *Noctilio leporinus*; and Phyllostomidae, *Phyllostomus discolor*), and two Yinpterochiroptera (Pteropodidae, *Rousettus aegyptiacus*; and Rhinolophidae, *Rhinolophus ferrumequinum*).

### Genome Sequencing

Samples for genomic analyses were acquired from museum collections (see Acknowledgements; supplementary table S1, Supplementary Material online). We selected samples recently collected and flash frozen in liquid nitrogen when available to maximize DNA quality. High-molecular-weight DNA was extracted using a commercial kit (MagAttract HMW DNA Kit, QIAGEN, USA, Cat No./ID: 67563). Genomic libraries and DNA sequencing were performed at the New York Genome Center (New York, USA) and GENEWIZ (New Jersey, USA) using a combination of 10X Chromium long-read and TruSeq libraries and HiSeqX and NovaSeq sequencers. The number of reads per genome ranged from 66 to 1,500 million reads, (average = 554 million), with a coverage of 37.1 to 46.6× (average = 42.5×) and estimated genome size of 2.38 to 3.34 Gbp (average = 2.78 Gbp; supplementary table S10, Supplementary Material online).

### Genome Assembly, Mapping, Repeat Modeling, and Masking

To generate genome assemblies, two approaches were followed depending on the library/sequencing method. Reference-guided assemblies were performed for reads generated by 10X Chromium. First, reads were assembled using Supernova v.2.1.1 (Weisenfeld et al. 2017). Then, contigs were mapped to the *M. myotis* genome published by Bat1K (Jebb et al. 2020) using minimap2 (Li 2018). For the rest of the genomes, reads were filtered (phred quality score ≥ 30) and then mapped to the reference genome using bwa (Li and Durbin 2009). Quality assembly metrics were calculated using assembly-stats v.563d5c3 (https://github.com/sanger-pathogens/assembly-stats). Unmapped reads were filtered, and then we used freebayes (https://github.com/freebayes/freebayes) to determine the most-likely genotype in a given genome for each position in the reference. Finally, consensus scaffolds were generated using bcftools consensus (Danecek and McCarthy 2017). The number of scaffolds ranged from 90 to 92, and the N50 ranged from 94.42 to 94.45 Mbp (average = 94.45 Mbp; supplementary table S2, Supplementary Material online). For further analyses and to prevent genome alignments from running too long due to repetitive regions, we generated de novo repeat libraries for each assembly using RepeatModeler (http://www.repeatmasker.org/, parameter -engine ncbi). The resulting libraries were then used to soft-mask the genome using RepeatMasker v.4.0.9 (parameters: -engine crossmatch -s).

### Pairwise Genome Alignments (Input for TOGA)

To generate pairwise genome alignments, we used LASTZ (Harris 2007) with parameters (*K* = 2,400, *L* = 3,000, *Y* = 9,400, and *H* = 2,000), and the LASTZ default scoring matrix that have a sufficiently high sensitivity to align orthologous exons between placental mammals (Sharma and Hiller 2017). Local alignments were chained using axtChain (Kent et al. 2003) with default parameters except for linearGap =loose). We used RepeatFiller (Osipova et al. 2019) with default parameters to add missed repeat-overlapping local alignments to the alignment chains and chainCleaner to improve alignment specificity using default parameters except for minBrokenChainScore = 75,000 and -doPairs (Suarez et al. 2017).

### Reference-based Gene Annotation Using TOGA

To generate gene annotations for further comparative analyses, we used an orthology-based approach as implemented in TOGA v. 9c196d5 (Kirilenko et al. 2023). TOGA is a machine learning–based method used to generate gene annotations based on exon-aware liftover from pairwise genome alignments between a reference species and a query; and jointly infer orthologue relationships (e.g. single-copy or multiple-copy genes). We used the human genome as a reference annotation (hg38, which includes 19,306 genes with 46,734 isoforms) and pairwise chain alignments generated as described above. Pairwise alignment chains used in TOGA can capture orthologous gene loci as well as loci containing paralogs or processed pseudogenes. To distinguish them, TOGA evaluates intronic and intergenic alignments and computes the “global CDS fraction.” High values indicate alignments overlapping coding exons (paralogous or pseudogene chains), while low values indicate alignments in intronic and intergenic regions (orthologous chains). Thus, only orthologous chains are used for gene projections. To assess the completeness of our reference-based genome annotations, we used BUSCO v.4.1.4 (Seppey et al. 2019) in protein mode with the mammalian protein set that consisted of 9,226 genes (mammalia_odb10). As an alternative metric of genome annotation quality, we evaluated the number of genes in the annotation with intact reading frames, inactivating mutations, fragmented or missing from the TOGA output.

### Exon-by-Exon Alignments

For comprehensive genome-wide selection screens, we generated exon-by-exon codon alignments for single-copy orthologous genes with intact reading frames, defined by TOGA as those with a unique copy and lacking inactivating mutations within at least the 80% central portion of the gene. Using the “extract_codon_alignment.py” script from TOGA (Kirilenko et al. 2023), codons with frameshifting insertions or deletions and premature stop codons were masked with “NNN.” Each orthologous exon was individually split and aligned using MACSE v.2 (Ranwez et al. 2018). All exons were then concatenated into a multiple-codon alignment. In addition, to identify and selectively remove poorly aligned sequence segments, codon alignments were cleaned with HmmCleaner (Di Franco et al. 2019) using default cost values. We retained gene alignments with a minimum of 20 sequences (60% of our target taxa) and at least one *Myotis* ecomorph per biogeographic clade (Fig. 1b), resulting in 16,426 genes. Excluded alignments with fewer than 20 sequences can result from non–single-copy orthologs in our annotations, encompassing duplicated genes, inactivating mutations near the N- or C-terminus, or missing genomic sequences due to assembly gaps.

### Genome-Wide Selection Screen and Gene Set Enrichment Analyses

To identify genes evolving under positive selection, we used an aBSREL method implemented in HYPHY (Kosakovsky Pond et al. 2005; Smith et al. 2015). aBSREL was run in exploratory mode to test all branches and nodes within a phylogenetic tree previously published for *Myotis* (Morales et al. 2019; supplementary fig. S6, Supplementary Material online). Those genes and branches with significant *P*-values after false discovery rate (FDR) correction across branches within each gene were retained. We explored if there was a correlation between the number of times a branch is found under selection and the number of times such a branch is represented in the gene alignments. We did not find a significant correlation (*R*² = 0.0022, *F*_1,29_ = 0.06432, *P* = 0.8016), indicating no bias in our results due to potential genome completeness heterogeneity (supplementary fig. S7, Supplementary Material online).

To explore if genes under positive selection in branches of interest (e.g. stem *Myotis* or those leading to biogeographic splits) tend to have a higher representation than expected by chance in specific pathways or GOs, we performed gene enrichment analyses using Metascape (https://metascape.org; Zhou et al. 2019). Multiple test corrections were applied as implemented in Metascape, providing FDR values as Log(q-value) with thresholds <5% to assess statistical significance. All genes annotated in the human genome (reported in version Ensembl 106) have been used as the enrichment background. We organized GOs into sets of classes with relations operating between them at different hierarchies. To focus on relevant GO terms, we set a threshold to highlight terms up to five levels below the primary parent term “Biological Processes,” with particular emphasis on developmental and cellular processes. To enhance visualization and contrast significantly enriched values, we plotted the negative logarithm of the *P*-value. Furthermore, to ensure that patterns of gene selection and enrichment for GO terms are specific to *Myotis* ecomorphs rather than reflecting general evolution within bats, we conducted a randomization test. We randomly selected the same number of genes as observed in each group in our dataset and performed enrichment analyses, contrasting results among *Myotis* ecomorphs and outgroup species. Moreover, we carefully explored gene enrichment results and the table of genes under selection in branches of interest, and we integrated knowledge from the literature while accounting for the relevance of genetic and biological function. Finally, to rule out that the gene enrichment patterns observed for each ecomorph are not driven by a single species, we performed enrichment analyses where each species is analyzed independently.

### Genome-Wide Convergence Assessment Among Foraging Strategies

To test if there is a correlation between the relative evolutionary rates of single-copy ortholog genes and the convergent evolution of each foraging strategy, we used a method that combines data transformation and weighted regressions while correcting for heteroscedasticity in gene evolutionary rates while accounting for phylogenetic relatedness, as implemented in RERconverge (Kowalczyk et al. 2019; Partha et al. 2019). Alignments for 16,426 genes were used to prune the species tree keeping only the taxa represented in each alignment. Such alignments were used to estimate branch lengths according to the best-fitted model (chosen by highest likelihood) for a given model of nucleotide substitution. Branch lengths were estimated using the R package “phangorn” (Schliep 2011). The relative evolutionary rates of each branch for the overall tree were estimated for all branches and each gene. We then estimated if there is an association between the variation of such evolutionary rates with a given foraging strategy, using binary tree traits (per foraging strategy) and the Kendall rank correlation coefficient, with a *P* < 0.01. For those genes with significant correlations, we performed gene enrichment analyses using Metascape and gProfiler2 (Kolberg et al. 2020), as described above.

To assess molecular convergence at the protein level within each gene, we employed the ωc metric implemented in CSUBST (Fukushima and Pollock 2023), a method designed to identify branch pairs exhibiting both convergent evolution and positive selection. The ωc contrasts the ratio of observed to expected non-synonymous combinatorial substitutions against the ratio of observed to expected synonymous combinatorial substitutions across distinct phylogenetic branches. We ran CSUBST four times for all single-copy ortholog genes in our dataset. Each time setting as foreground branches those for the three *Myotis* ecomorphs, and one in exploratory mode without foreground branches. To designate a pairwise branch comparison as convergent, we retained genes and branch combinations with ωc values ≥3 for any ancestral state transitioning to specific derived states observed in our terminal branches with known foraging strategies (“any2spe”).

Finally, to identify genes with a statistically significant signal of positive selection, convergent relative evolutionary rates of nucleotide substitution, and molecular signatures of protein convergence, we overlapped the genes identified with aBSREL, RERconverge, and CSUBST with significant *P*-values or ωc ≥ 3 and performed enrichment results as described above.

### Divergence Time and Node Age Estimation

To estimate the ages of nodes and associate them with probabilities of gene reuse, we first used a penalized likelihood approach implemented in treePL (Smith and O’Meara 2012) to determine the divergence times among lineages. We conducted two analyses—the first to optimize the parameters for treePL and the second using the optimized values. Fossil calibrations were used in both runs to constrain the maximum divergence times at relevant nodes. Although more comprehensive time-calibrated phylogenies have been estimated for *Myotis* and other bats (e.g. Agnarsson et al. 2011; Morales et al. 2019), we required pairwise estimations for all taxa in our phylogeny, and thus, we performed independent calculations. Our estimated values were very similar to previously reported values. To calculate node age estimates for each species pair, we summed the branch lengths from each lineage pair to their most recent common ancestor and divided them by two.

### Approximating the Proportion of Gene Reuse Linked to the Convergent Evolution of Foraging Strategies

To investigate the relationship between the proportion of shared genes under positive selection (identified with aBSREL) and the divergence time (node age) for each species pair with convergent phenotypes, we applied a framework following Conte et al. (2012). Such an approach initially focused on genes with validated experimental links to convergent phenotypes and estimated the probability of gene reuse for lineages displaying convergent traits. While previous studies have primarily focused on genes with established experimental associations, our study represents a pioneering approach by leveraging genomic screens to identify candidate genes in *Myotis* bats potentially linked to the convergent evolution of ecomorphs. Although experimental validations directly linking these genes to phenotype evolution are currently lacking, we capitalize on our comprehensive genomic dataset to establish a null expectation for our model. Thus, we first estimated the correlation coefficient between all shared genes under positive selection or with molecular signatures of convergence and the divergence time among all the species pairs (terminal branches). Such analysis could not be performed for genes identified with convergent relative evolutionary rates by RERconverge because the output of such analyses represents a summary of the entire gene, not for each pair of branches within the gene.

Finally, to estimate gene reuse linked to phenotypic evolution, first, we identified sets of candidate genes associated with critical biological processes, such as cell morphogenesis (GO: 000090) and skeletal system development (GO: 0001501), based on significant results from gene enrichment analyses within *Myotis*. Importantly, these associations were not observed in outgroup species, highlighting the specificity of our findings to the *Myotis* lineage. Then, we fitted linear models to estimate if there is a relationship between node age and the proportion of genes under selection or with molecular signatures of convergence in our candidate sets. To calculate relative proportions of gene reuse, we divided the number of shared genes in each pair by the maximum number of observed shared genes between *Myotis davidii* and. *Myotis brandtii*, which share 103 genes under selection; thus, this species pair shows a value of 1.0. We did not use the total number of genes under selection as a maximum value for the transformation because the denominator is too big, and the differences among values are so minor that the resulting values would require an additional log-scale transformation for interpretation.

## Supplementary Material

msae165_Supplementary_Data

## Data Availability

Data and scripts relevant for this project are available at https://github.com/ariadnamorales/2023_Myotis_ConvergenceGenomics. Genomes and annotations are available at the Senckenberg mirror of the UCSC genome browser at https://genome.senckenberg.de.
