# Supplementary material for: Distinct Genes with Similar Functions Underlie Convergent Evolution in Myotis Bat Ecomorphs
Source: Mol Biol Evol. 2024 Aug 8;41(9):msae165. doi: 10.1093/molbev/msae165 (PMC11371419; doi:10.1093/molbev/msae165)

# SUPPLEMENTARY MATERIAL

**Distinct Genes with Similar Functions Underlie Convergent Evolution in *Myotis* Bat Ecomorphs**

Ariadna E. Morales, Frank T. Burbrink, Marion Segall, Maria Meza, Chetan Munegowda, Paul Webala, Bruce D. Patterson, Vu Dinh Thong, Manuel Ruedi, Michael Hiller, Nancy B. Simmons

#

# SUPPLEMENTARY FIGURES

[**Supplementary Figure 1.** Annotation statistics based on the percentage of completeness according to BUSCO.](#_3znysh7)

[**Supplementary Figure 2.** Annotation stats based on the number of genes with intact reading frames as projected by TOGA.](#_3dy6vkm)

[**Supplementary Figure 3.** Enrichment results of genes under selection in Myotis ecomorphs and outgroup species.](#_1t3h5sf)

[**Supplementary Figure 4.** Enrichment results of genes under selection in each Myotis species for gene ontologies in which ecomorphs (with aggregated species) also show significant results.](#_tn9n2q7sum98)

[**Supplementary Figure 5.** Myotis species represented in our datasets compared to former phylogenetic studies .](#_9vrpgs5v3stb)

[**Supplementary Figure 6.** Phylogeny with branch labels used as input for gene selection analyses in aBSREL.](#_2s8eyo1)

[**Supplementary Figure 7.** No correlation between the number of times a branch is present in gene alignments and the times are found under selection according to aBSREL.](#_yyi7juig2llt)

#

# SUPPLEMENTARY TABLES (See attached Excel file)

- **Supplementary Table 1.** Information of genomes included in the genomic screen.
- **Supplementary Table 2.** Genome assembly statistics for all *Myotis* genomes in this study.
- **Supplementary Table 3.** Genes and branches with a statistically significant signal of positive selection in a screen including 22 *Myotis* and 8 outgroup species.
- **Supplementary Table 4.** Genes with a statistically significant selection signal per ecomorph.
- **Supplementary Table 5.** Enrichment results from Metascape using as input list of genes under selection in at least one branch per *Myotis* ecomorph, values represent -Log(q-value) after multiple test corrections.
- **Supplementary Table 6.** Results of RER converge analyses showing genes with significant signal of convergent evolutionary rates per ecomorph.
- **Supplementary Table 7.** Enrichment results from Metascape for gene ontologies related to biological processes, using a list of genes showing significant signal of convergent evolutionary rates within ecomorphs as identified by RERconverge,values represent -Log(q-value) after multiple test corrections.
- **Supplementary Table 8.** Results of csubst analyses showing genes with significant signal of protein convergence in at least 2 species per ecomorph.
- **Supplementary Table 9.** Enrichment results from metascape for gene ontologies for biological processes using as input list of genes with significant signal of protein convergence within ecomorphs according to csubst, values represent -Log(q-value) after multiple test corrections.
- **Supplementary Table 10.** Sequencing and assembly methods for new *Myotis* genomes.

## Supplementary Figure 1. Annotation statistics based on the percentage of completeness according to BUSCO.

BUSCO was applied to protein sequences of annotated genes by TOGA. Bar charts represent the percent of highly conserved mammalian BUSCO genes (mammalia_db10) that are found to be complete (gray), fragmented (yellow), or missing (red) in the assembly. Labels correspond to species names and, in parentheses, the Senckenberg genome browser ID assigned to the assembly and annotation—more information in Supplementary Table 1.


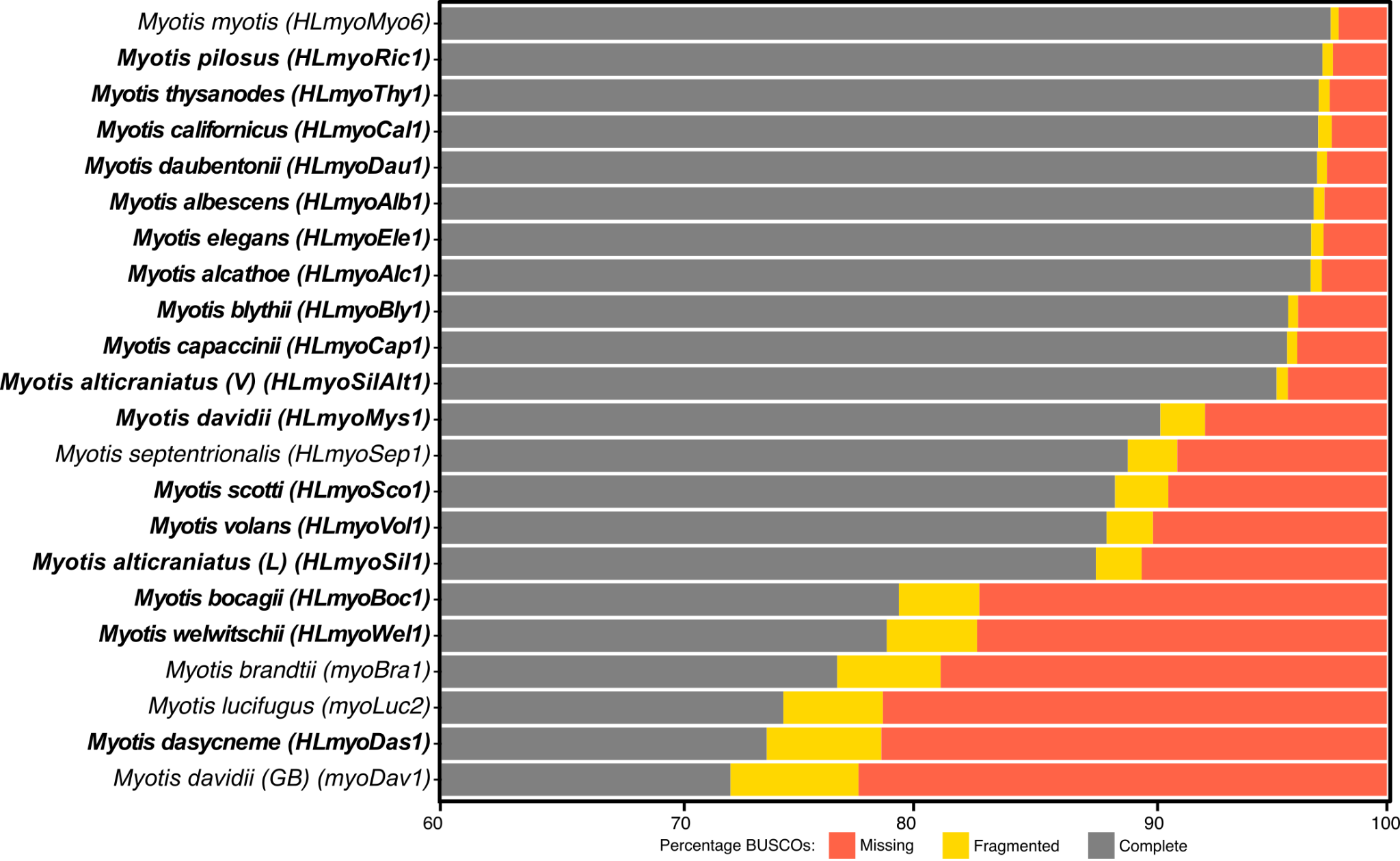


##

##

## Supplementary Figure 2. Annotation stats based on the number of genes with intact reading frames as projected by TOGA.

Status of 18,430 ancestral mammalian genes in 22 *Myotis* genomes used for genomic screen. Ancestral genes are classified by TOGA (using human hg38 as the reference) into those with an intact reading frame (blue), gene-inactivating mutations (premature stop codons, frameshifts, splice site disruptions, and deletions of exons or entire genes; shown in orange) or missing or incomplete coding sequences due to assembly gaps or fragmentation (gray). An excess of genes with missing sequences indicates a lower assembly completeness and an excess of genes with inactivating mutations indicates a lower base accuracy. The seventeen newly-generated genomes are shown in bold font. Assemblies are sorted by the number of intact genes.


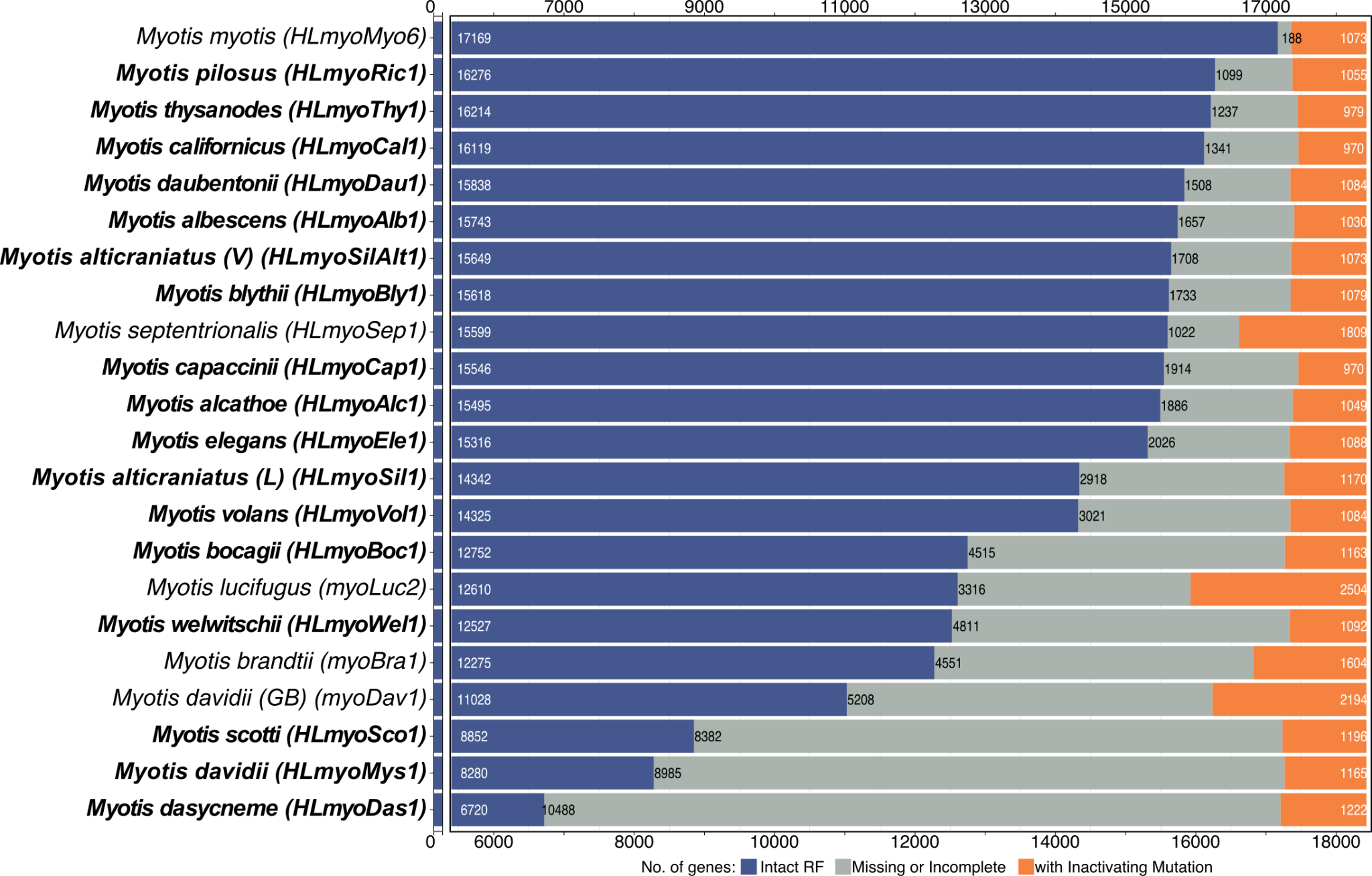


## Supplementary Figure 3. Enrichment results of genes under selection in Myotis ecomorphs and outgroup species.

We compare results between observed and randomly selected genes showing that selection among genes involved in cellular and developmental processes in Myotis ecomorphs is not the result of general background evolution among all bats.


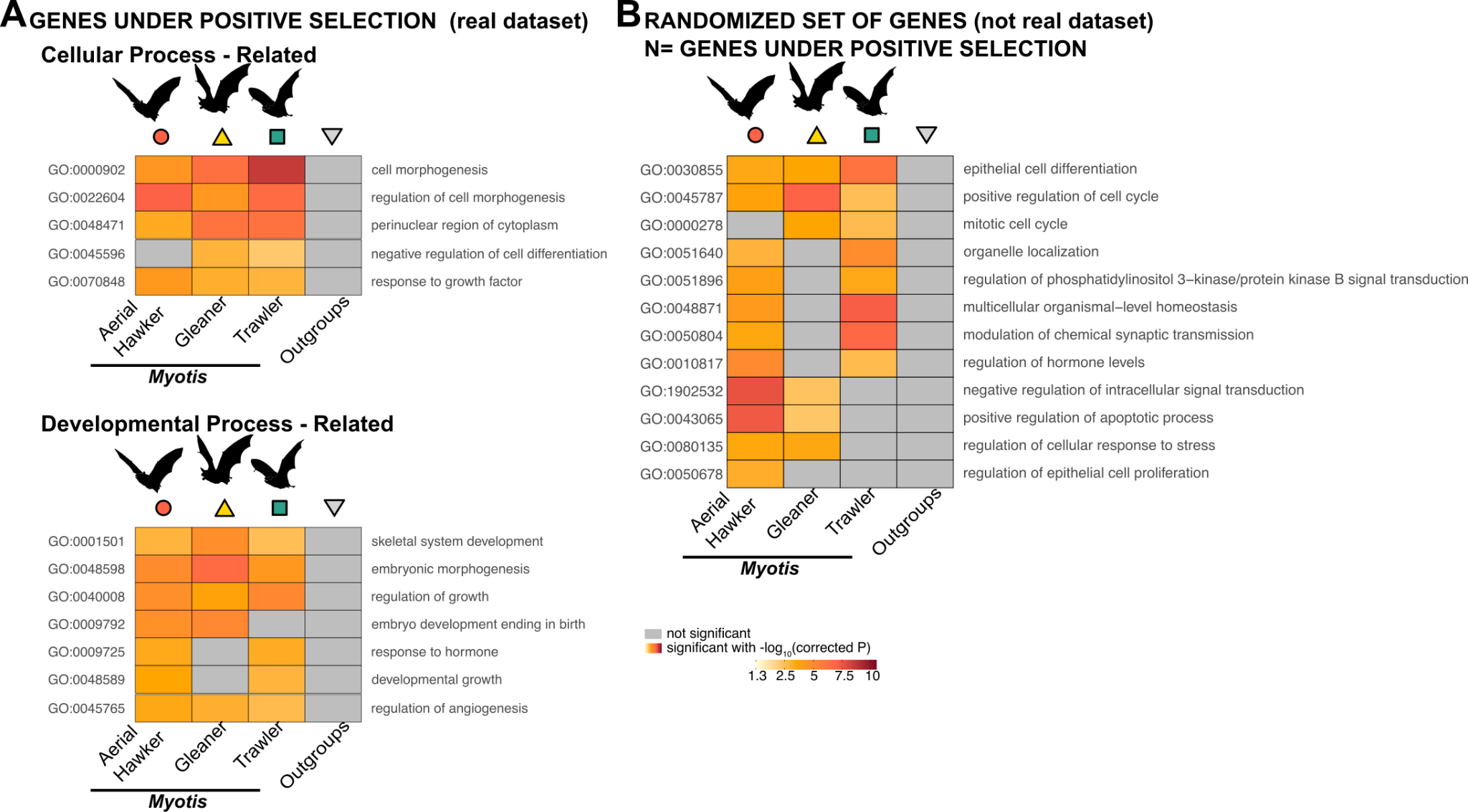


## Supplementary Figure 4. Enrichment results of genes under selection in each *Myotis* species for gene ontologies in which ecomorphs (with aggregated species) also show significant results.

Specific (second- or third-level) gene ontologies not shown.


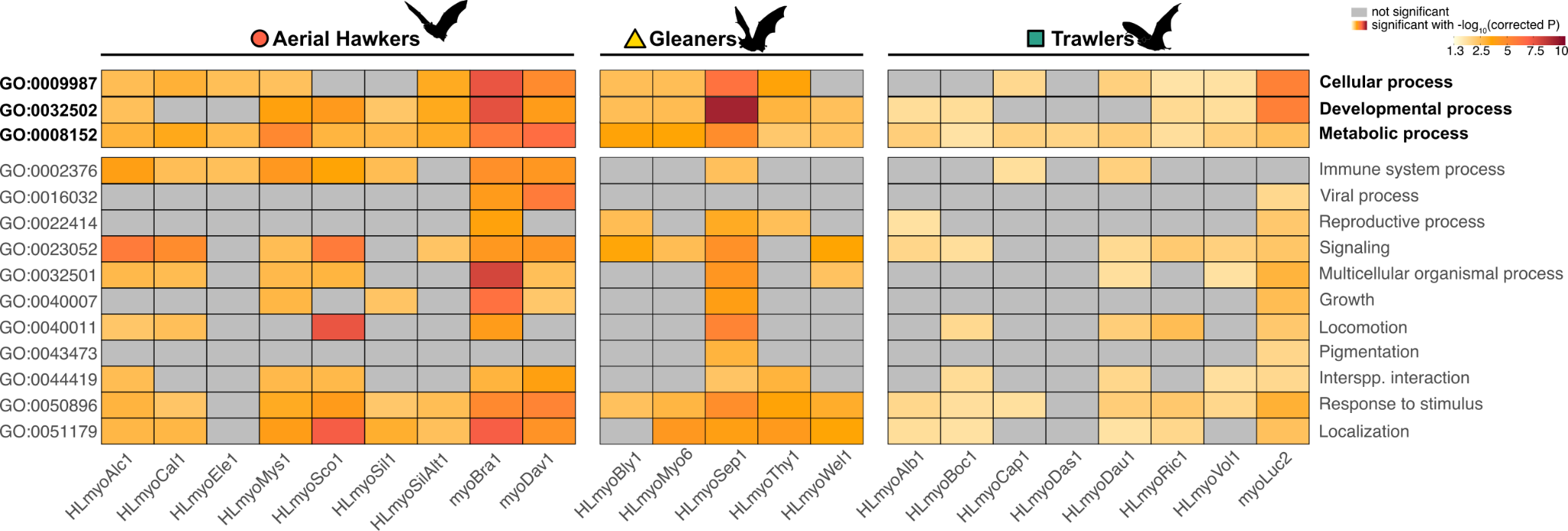


##

## Supplementary Figure 5. *Myotis* species represented in our datasets compared to former phylogenetic studies.

Comparison of taxonomic coverage between previous phylogenetic studies including ~80% of extant *Myotis* species and partial genomic data (Morales et al. (2019) and the dataset in this study comprising 20 whole genomes of *Myotis* species highlighted with bold fonts. Symbols at the terminal branches indicate foraging strategy as trawler (square), aerial hawker (circle), or gleaner (triangle).


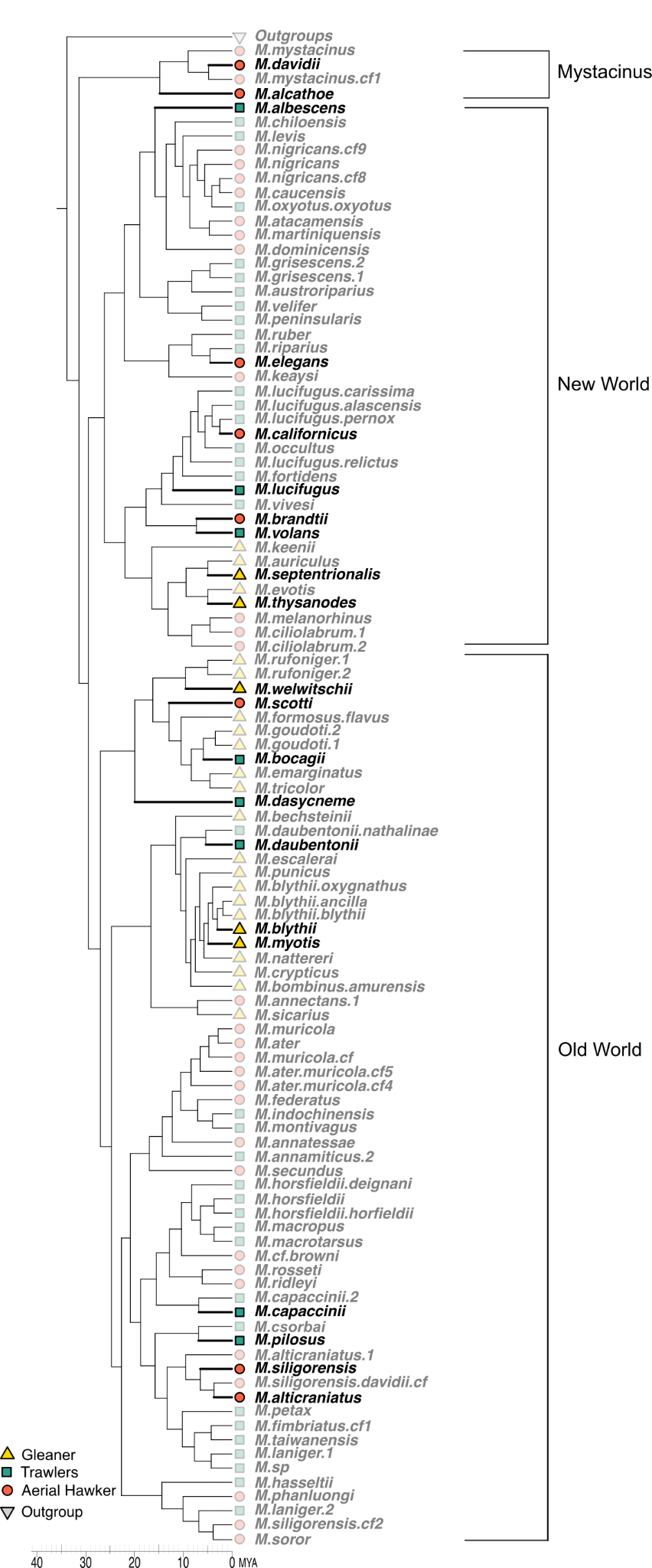


## Supplementary Figure 6. Phylogeny with branch labels used as input for gene selection analyses in aBSREL.

Branch labels correspond to “Senckenberg Genome Browser ID” shown in Supplementary Table 1. Internal node labels show IDs used for screen in aBSREL and correspond to selection results shown in Supplementary Table 3. At the terminal branches, symbols on the first row indicate foraging strategy as trawler (square), aerial hawker (circle), or gleaner (triangle). Hexagons in the second row indicate biogeographic regions and species distribution colored following their map at the lower left, and labeled as Nearctic (Nr), Western Palearctic (WP), Eastern Palearctic (EP), Afrotropical (Af), Neotropical (Nt), Indomalayan (IM), and Oceanian (Oc). All branch lengths have the same length and do not represent any evolutionary process.


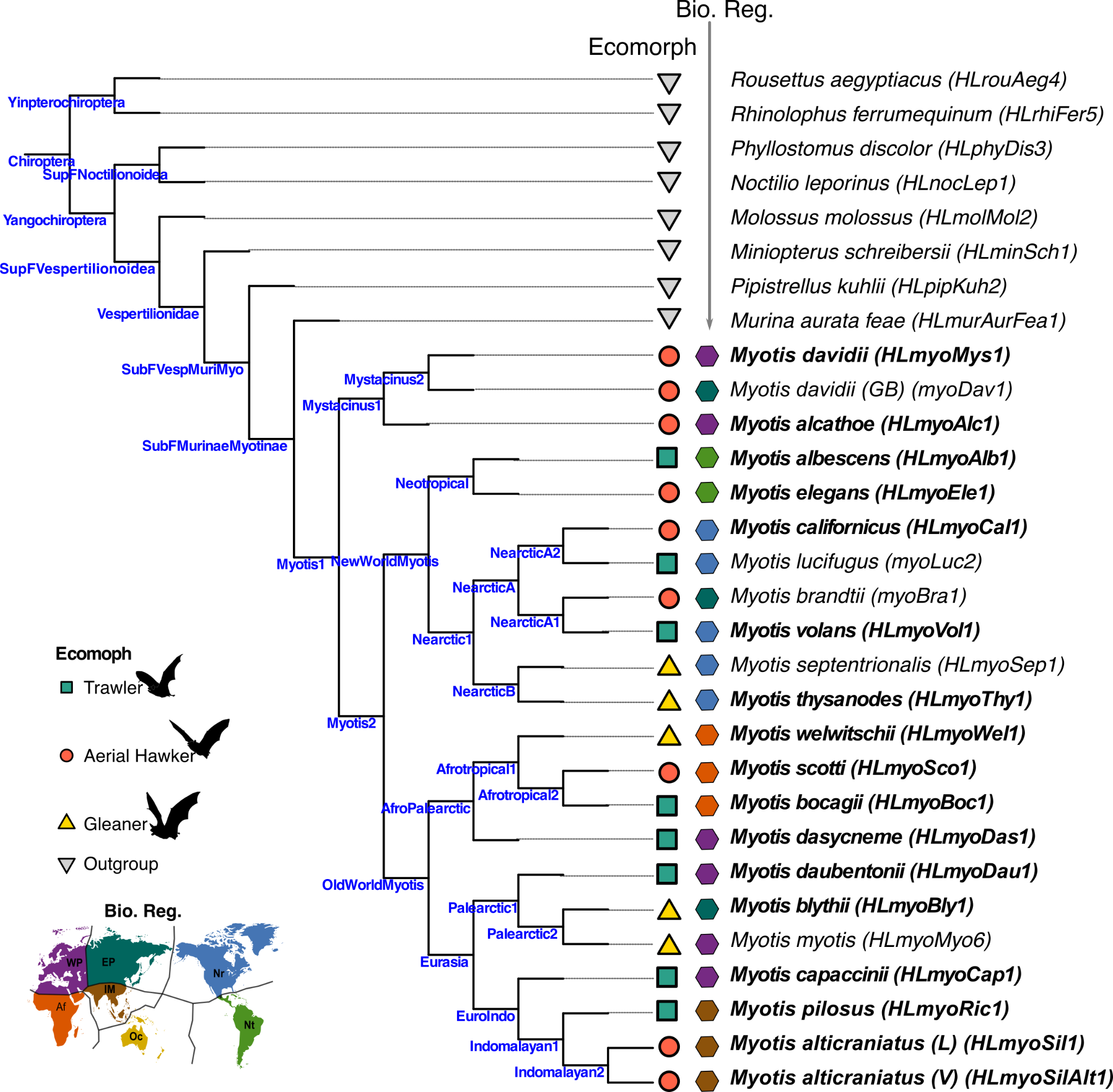


## Supplementary Figure 7. No correlation between the number of times a branch is present in gene alignments and the times are found under selection according to aBSREL.

Points represent *Myotis* and non-*Myotis* species.


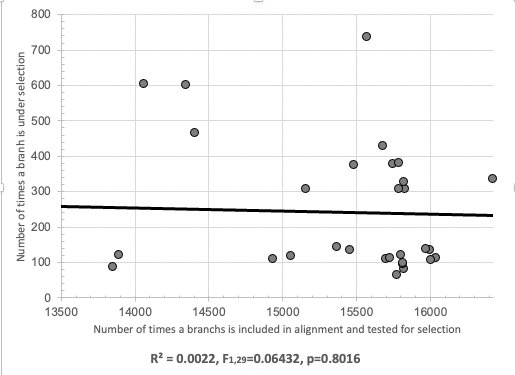

Supplement: msae165_Supplementary_Data [file msae165_supplementary_data.zip › R2_MBE.GENOMICS_SM-ConvMyotis_v1.docx]
